# Efficacy of a multi-nutrient dietary supplement on improving decision fatigue in video gamers

**DOI:** 10.3389/fnut.2025.1680030

**Published:** 2025-11-21

**Authors:** Bandana Seesurn, Rodrigo Batllori, Shawn N. Watson

**Affiliations:** Senescence Life Sciences Pte. Ltd, Singapore, Singapore

**Keywords:** decision fatigue, decision-making, cognitive performance, e-sports, cognitive function, dietary supplement

## Abstract

**Introduction:**

Gamers can experience decision fatigue, affecting decision-making and overall performance. This pilot study evaluated a multi-nutrient supplement called Numin on decision fatigue, video game performance, cognitive function, and mouse and keyboard input mechanics assessed as physical aspects of video gamers. Formulated with active ingredients like Methylsulfonylmethane, L-Tyrosine, Rhodiolife^®^, TurmiPure Gold^®^, and Chromium Picolinate, Numin was examined in a randomized, placebo-controlled, crossover study.

**Methods:**

Healthy adults with at least “Silver” skill rating were enrolled from February 6 to 13, 2022 in Arizona, USA. Participants played 10 competitive games of League of Legends (LoL). The primary endpoint was the change in decision fatigue between study groups, measured using Decision Fatigue Scale (DFS) and video game performance.

**Results:**

DFS scores were lower in the intervention group than in the placebo group, while cognitive assessment scores were higher in the intervention group for most tasks compared to the placebo group. However, these differences were not significant. Video game performance significantly improved in the intervention group regarding average deaths when losing, gold spent percentage difference, and perhaps most importantly, win rates compared to the placebo group (*p* < 0.05 for all). While the intervention group showed no significant difference in chat cycles or champion recall, the placebo group demonstrated significant reductions in both, indicating that the intervention group maintained performance in these aspects relative to the decline in the placebo group. The intervention group showed significant changes in the number of mouse clicks and mouse movements, compared to the placebo group.

**Discussion:**

Numin is hypothesized to exert neuroprotective effects by improving glutamate re-uptake and protecting against excitotoxic injury, thereby supporting neuronal health and neurotransmission. While direct measures of decision fatigue and cognition did not show statistically significant improvements, these mechanisms may contribute to the observed enhancements in gaming performance and win rates by mitigating the molecular and cellular events underlying cognitive decline under prolonged demands. Our findings indicate that Numin improved video game performance in LoL gamers, suggesting it may support cognitive function during intense tasks. This study highlights the potential application and benefits of such a supplement in individuals undergoing cognitively intense tasks, such as gaming.

## 1 Introduction

The neurological basis of decision-making is multifaceted and complex, involving various neurotransmitter signaling systems. Glutamatergic signaling is the primary excitatory signaling system in decision-making, with dopaminergic and serotonergic signaling playing crucial roles as well (1–3). Research shows that individuals engaged in cognitively demanding tasks often experience decision fatigue, which is characterized by the progressive decline in the ability to make high-quality decisions following a period of consecutive decision-making (4–6). As a result, individuals are more likely to use heuristics rather than deliberate reasoning when making decisions (6, 7). This often leads to the selection of simpler choices that may not be optimal for the situation, and in severe cases, can result in decision avoidance altogether (6, 7). Consequently, decisions made under such conditions can be regarded as impulsive or irrational and pose significant implications in high-pressure environments where effective decision-making is critical (6–8).

League of Legends (LoL) is a popular videogame within the Multiplayer Online Battle Arena (MOBA) genre, where two teams of five players compete to destroy the opposing team's base using unique characters, called champions, with special abilities (9, 10). Throughout a game, teams accumulate resources called ‘gold' and ‘experience points' from various actions like completing objectives (i.e., destroying towers), killing players from the opposing team, and killing computer-controlled creatures (10). These resources can be used to purchase items or upgrade special abilities, providing champions with advantages over the opposing team (10). The fast-paced, competitive, and dynamic nature of LoL requires players to rapidly analyse the risks associated with their actions while simultaneously responding to the opposing teams' actions (11). This creates a high-pressure environment where players make continuous real-time decisions throughout each game, which can last between 20 and 60 min (11, 12). Decision fatigue is cited as a contributing factor to a condition referred to as ‘tilting', which is a major cognitive disabler for gamers (13).

As there are no available treatments or interventions that specifically target decision fatigue, gamers often rely on stimulants such as caffeine and pharmaceutical products (i.e., modafinil) to enhance gaming performance and delay fatigue (14, 15). While studies found some performance improvements with traditional stimulants, the degree of improvement varies. For instance, studies on caffeine report decreased reaction times by 5–46%, while others show no statistically significant improvements (14, 16, 17). Worryingly, stimulants like caffeine can lead to negative effects, such as increased blood pressure and poorer sleep quality, which in turn can cause anxiety, jitteriness, and impulsivity, all of which can negatively impact gaming performance (14). This has garnered rising interest in using natural-based compounds to enhance mental and cognitive function during gaming. As such, this pilot study aimed to determine the efficacy of a multi-nutrient dietary supplement (Numin) on decision fatigue, cognitive function, video game performance, and physical aspects in video gamers over a 13-h period. The primary endpoint of this study was the perceived change in decision fatigue intensity, measured using Decision Fatigue Scale (DFS) and overall gameplay performance, between the intervention and placebo groups. The secondary endpoints were the changes in cognitive function measured using Creyos cognitive assessment, and physical aspects measured through mouse input mechanics between the study groups. The active ingredients in Numin including Methylsulfonylmethane, L-Tyrosine, Rhodiolife^®^ (Rhodiola rosea (roots), total rosavins ≥ 5%, salidroside ≥ 1.8%), TurmiPure Gold^®^ (with over 30% curcuminoids), and Chromium Picolinate, are hypothesized to contribute to the study's outcomes through various mechanisms. For instance, curcumin and *Rhodiola rosea* root extract are recognized for their neuroprotective properties, potentially enhancing glutamate re-uptake and safeguarding against excitotoxic injury. By supporting neuronal health and neurotransmission, these ingredients may contribute to improved cognitive function and resilience against decision fatigue. Furthermore, other active ingredients like methylsulfonylmethane, L-tyrosine, and chromium picolinate are also believed to play a role in preserving and enhancing neurotransmissions vital for decision-making processes.

Specifically, improved glutamate re-uptake and protection against excitotoxic injury could lead to sustained neural function. By maintaining a healthier neuronal environment, the supplement may help prevent subtle, yet impactful, declines in neural efficiency that occur during prolonged cognitive tasks, which could translate to more consistent and effective processing of in-game information. Additionally, enhanced neurotransmission and neuronal health could support faster and more accurate signal processing, potentially leading to improved reaction time and precision. In a fast-paced game like League of Legends, even small enhancements in these areas could impact performance metrics such as average deaths when losing, champion recalls, chat cycles, and gold spent percentage difference. Moreover, these neuroprotective effects might provide a subtle but effective buffer against the cumulative cognitive load of competitive gaming, potentially allowing players to maintain a higher level of performance for longer, and thereby contributing to improved win rates. This suggests that these neurochemical supports may contribute to a sustained higher level of performance during cognitively demanding tasks.

## 2 Materials and methods

### 2.1 Study design

This double-blinded, randomized, placebo-controlled, crossover study was conducted at an e-sports center (Gilbert, Arizona, USA) to determine the efficacy of a multi-nutrient dietary supplement (Numin) on decision fatigue, cognitive function, video game performance, and physical aspects in video gamers. Numin supplement's active ingredients included Methylsulfonylmethane, L-Tyrosine, Rhodiolife^®^ (Rhodiola rosea (roots), total rosavins ≥ 5%, salidroside ≥ 1.8%), TurmiPure Gold^®^ (with over 30% curcuminoids), and Chromium Picolinate. The inactive ingredients consist of Xantham Gum, Beet Powder, Stevia, Citric Acid, and Natural Orange Passionfruit Flavor. The study aimed to enroll up to thirty participants in this two-study arm crossover design, however, twenty-three participants were recruited locally and enrolled in the study. The sample size was determined with a 90% probability of detecting a study arm difference at a two-sided 0.05 significance level. This calculation was based on the assumption that the within-patient standard deviation of the response variable is 3.11 and the true difference between study arms is 2.699 units. These values were derived from previous literature examining the short-term cognitive effects of the active ingredients within the product. Twenty-three participants were considered a suitable number. In this design, each participant acted as their own control, experiencing both the intervention and placebo conditions. This approach significantly enhanced statistical power by reducing inter-individual variability, making the effective sample size for within-group and intervention-placebo comparisons effectively 23 participant-observations per condition. The participants met all the inclusion criteria and none of the exclusion criteria. Participants were randomly assigned to either the investigational product or placebo arm on Day 1, followed by assignment into the opposite arm on Day 8. The active study duration was 2 days for each study participant.

**Figure 1:**
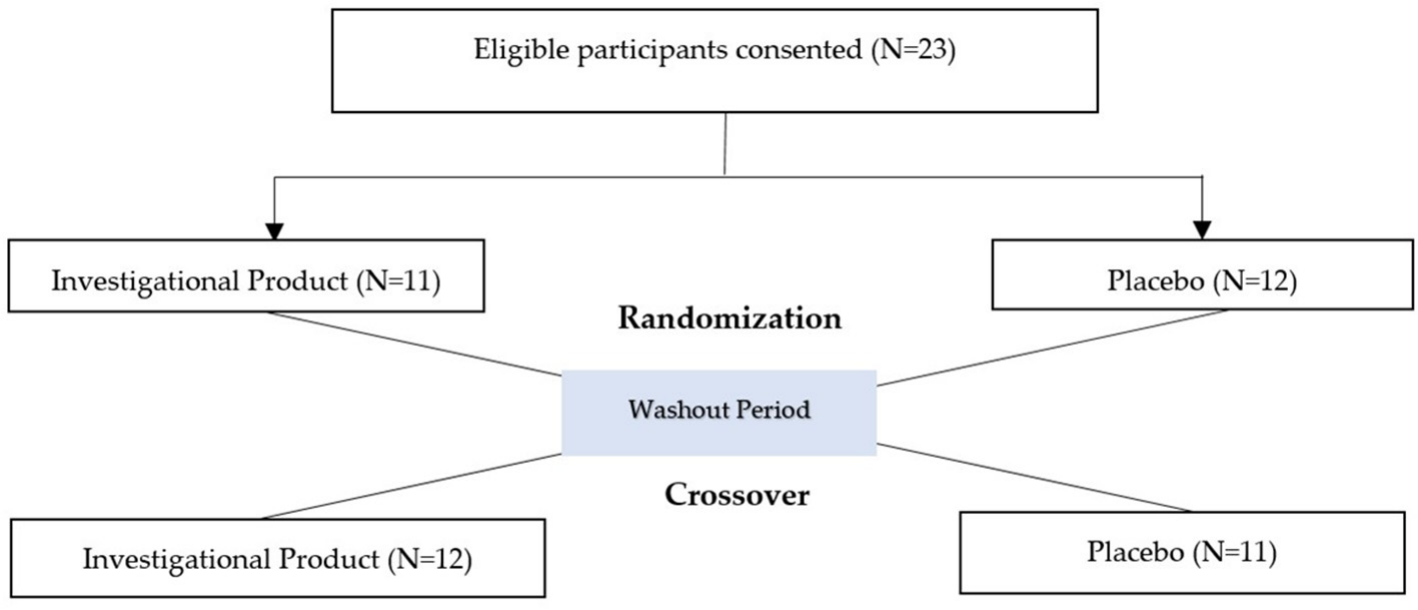
Study design.

Figure 1 provides a flowchart depicting the study's crossover design.

The study protocol was conducted in accordance with the Declaration of Helsinki and approved by the Institutional Review Board of Advarra (Pro00056208) on 29 July 2021. The study was conducted in accordance with the International Conference on Harmonization (ICH) and Good Clinical Practice (GCP) guidelines. Informed consent was obtained from all subjects involved in the study.

### 2.2 Participants

The study was conducted in Pheonix, Arizona, USA. Recruitment of video gamers was conducted from September 1, 2021, to February 1, 2022, and was done through social media (Instagram and Facebook) and local poster advertisements in gaming cafés. Selection of participants was based on the following inclusion criteria: (a) were healthy, (b) were above the age of 18 years, (c) had completed secondary school or higher-level education, and (d) had an in-game skill rank level of either “Silver” or above. The inclusion criteria of the study focused on healthy adults, which typically implies an absence of ongoing chronic conditions requiring such medications. Participants were excluded from the study if they were currently taking any prescription medications known to affect cognitive function, energy levels, or central nervous system activity. This included, but was not limited to, medications such as naltrexone, which has been associated with changes in fatigue levels in certain populations. Additionally, participants taking any supplements, vitamins, or minerals were excluded, with a 14-day washout period required prior to screening for supplements and over-the-counter medications, or as assessed and determined by the Principal Investigator (PI). Eligible participants had to agree to: (a) abstain from caffeine-containing foods or beverages for 48 h prior to study visits, (b) refrain from nicotine-containing products during study visits and (c) avoid ingesting more than 36 g of added sugars per day during study visits. This recommended intake of 36 grams per day aligns with the guideline for adults from the American Heart Association (AHA). “Added sugar” referred to sugars and syrups added to foods or beverages during processing or preparation, and not naturally occurring sugars found in fruits or milk. Adherence to this dietary restriction was controlled through a diet log maintained by the participants. This dietary restriction was put in place because, for gamers, consuming added sugar can lead to a short-term boost in energy and focus, followed by a “sugar crash” that impairs concentration, mental processing, and overall video game performance. Eligible study participants also agreed to having a minimum of 6 h of sleep on the eve of study visits, monitored through a sleep log. Participant informed consent forms were signed and collected prior to commencement of the study.

### 2.3 Study protocol

**Figure 2:**
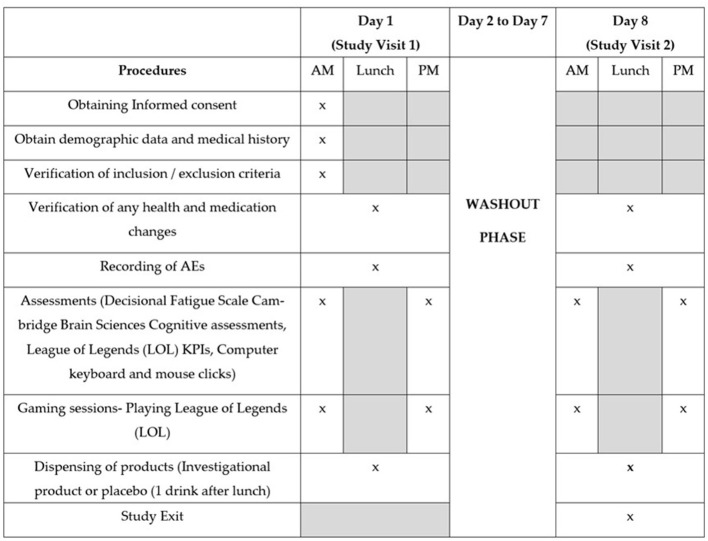
Study plan and timing of procedures.

On Day 1 (Study Visit 1), participant demographic and medical history data were recorded. Participants were randomized to the study or placebo group in a 1:1 ratio and completed the DFS and Creyos cognitive assessment to establish decision fatigue and cognitive function baselines. Participants played LoL against random skill-matched players determined by the in-game online ranking system (Ranked Solo Queue). Each participant played three games of LoL until the 30-min lunch break, where either the intervention (Numin) or placebo was administered. Five more games of LoL were played until the 30-min dinner break, followed by an additional two more games. After the final game of Study Visit 1, participants completed the DFS and Creyos cognitive assessment again and were discharged from the study site. From Day 2 to Day 7, participants underwent a washout phase. On Day 8 (Study Visit 2), participants entered the other study arm. Participants underwent the same schedule of recording baselines, gaming sessions, breaks, and reassessment of cognitive function and decision fatigue (see Figure 2 for the study plan and timing of procedures). A study exit form was filled out by each participant before completing the study. Throughout this study, all gameplay metrics were recorded, alongside mechanical inputs from each participants' keyboard and mouse.

### 2.4 Intervention

The constituents of the intervention (Numin) and placebo used in this study can be found in Appendix A -Table A1. The intervention and placebo were provided to blinded participants in powdered sachets that matched in color, appearance, flavor, and weight. The sachet containing the intervention or placebo was mixed with 355 mL of room temperature water in a bottle and was given to participants to consume on site within an hour of being dispensed.

### 2.5 Measurements

#### 2.5.1 Decision Fatigue Scale (DFS)

The DFS, originally described by Hickman et al. (18), was used in this study to provide quantitative measurements to the perceived decision fatigue of participants. The DFS is a subjective measure of decision fatigue consisting of a series of questions that measure the emotional, cognitive, and behavioral effects of decision fatigue, including emotional distress, mental exhaustion, and impulsive decision-making (18). Participants endorsed the scale items on a 4-point Likert scale from 0 (strongly disagree) to 3 (strongly agree). A total DFS score was derived by summing participants' response for each question.

#### 2.5.2 Creyos cognitive assessment

The Creyos cognitive assessment is a series of computer-based assessments used to measure cognitive function in participants. Participants completed nine tasks that assessed reasoning, memory, attention, and verbal activity. These tasks include: ‘Double Trouble', ‘Digit Span', ‘Feature Match', ‘Polygons', ‘Monkey Ladder', ‘Rotations', ‘Spatial Span', ‘Token Search', and ‘Spatial Planning'.

#### 2.5.3 Keyboard and mouse inputs

A freeware application, WhatPulse, was used to monitor and track participants' keyboard and mouse inputs. For keyboard inputs, this study tracked the number of times participants communicated with their teammates using the chat function (chat cycles), communicated with teammates using the visual alert system (pings), retreated to their team base (champion recalling), activated summoner spells (‘D' or ‘F'), used champion abilities (‘Q', ‘W', ‘E', or ‘R'), and checked the in-game scoreboard (‘tab'). These values were expressed as a percentage of the total number of keyboard strokes. For mouse inputs, the total number of mouse clicks, total mouse movement, variation in mouse movement, and mouse distance between clicks were recorded for each participant.

#### 2.5.4 Video game performance

Due to LoL's competitive nature and global adoption as a spectator event, rigorous metrics have been developed to measure performance. The performance metrics analyzed in this study are categorized into game outcomes, early-game performance, and overall player performance.

##### 2.5.4.1 Game outcomes

A player's win rate is defined as the proportion of wins to total games played and can be an indicator of player and team performance. In this study, the win rates of participants across the 10 games were recorded, and the group average was normalized to their respective group baseline levels recorded during the morning session (normalized win rate). To evaluate how decisively participants from each group won or lost their games, individual gold spent percentage difference (GSPD) was recorded across the 10 games and the group average was determined. This metric calculates the average percentage difference of gold spent at the end of each game between the two teams, which can indicate the extent of a participants' team victory or defeat (win-loss margin).

##### 2.5.4.2 Early-game performance

As a game progresses, players often group up and traverse the map as a team, making it difficult to evaluate individual contributions. Therefore, the early-game phase (initial 15 min) is suitable for measuring individual skill and performance. This study assessed early-game performance by comparing differences in (a) creep scores, the number of minions or neutral camps (computer-controlled creatures) killed; (b) gold, the amount of gold accumulated; and (c) experience points, the amount of experience points accumulated, between participants and their lane opponents (opposing team players occupying the same positions on the map). This data was captured for each participant at the 10 and 15-min marks of each game, and a group average was determined. All early-game metrics were contextualized using difference-from-expected values, as early-game metrics are heavily influenced by roles played (‘Top Lane', ‘Middle Lane', ‘Attack-Damage-Carry', ‘Jungle', and ‘Support') and champions selected.

##### 2.5.4.3 Overall player performance

To assess each players overall performance, three parameters were measured throughout the 10 games including: (a) average amount of gold earned per minute (gold per minute); (b) average amount of minions or neutral camps killed per minute (creep score per minute); and (c) average amount of damage delt to opposing team players per minute (damage to champions per minute). The player's total kills, assists, and deaths were also recorded at the end of each game. A group average was determined for the data collected.

### 2.6 Safety assessment

Continuous monitoring of adverse events (AEs) and serious adverse events (SAEs) was done throughout this study, and the trial adhered to the United States Food and Drug Administration (US-FDA) requirements for the reporting of such events. No adverse events (AEs) or serious adverse events (SAEs) were reported among the participants in either the intervention or placebo group throughout the duration of the study.

### 2.7 Statistical analysis

Statistical analysis was performed using Statistica Version 14. Descriptive statistics for participant characteristics was presented as mean and standard deviation. Inferential statistics, including repeated measures ANOVA, paired *t*-test, and independent *t*-test were performed on data sets which met all requirements for normality and variance and were appropriately selected to leverage the power benefits of the crossover design of the study. This inherent characteristic of a crossover design significantly enhances the statistical power compared to a parallel-group design with the same number of participants, as it reduces inter-individual variability and allows for more precise comparisons. Log-transformed data was used when normality requirements were not met due to variability in datasets and was only used on video game performance data. Video game performance data was excluded if a participants' results exceeded three standard deviations outside of the mean. *Post-hoc* comparison was used to garner additional information about effect size. A *p*-value < 0.05 indicates statistical significance for all tests.

## 3 Results

### 3.1 Participant characteristics

Twenty-three participants (22 males, 1 female) completed the study, with no AEs or SAEs reported among the participants in either the intervention or placebo group throughout the duration of the study. The average age of participants was 27.7 years (standard deviation 5.4 years). All participants were competent at the game, having a LoL account level of at least 30 and a minimum skill level group of “Silver” in the “Ranked Solo Queue” mode.

### 3.2 Decision fatigue scale (DFS) scores

The placebo group had a marginally higher overall mean DFS score compared to the intervention group, but this difference was not statistically significant (Table 1).

**Table 1:** DFS scores of the intervention and placebo group for each question.

	**Intervention group**	**Placebo group**	***p*-value**
**Overall mean DFS scores**	7.4 ± 6.5	7.8 ± 6.3	0.70

*p*-value from a test of difference in proportion with an independent t-test. Data is presented as mean ± standard deviation.

### 3.3 Creyos cognitive assessment scores

No significant differences were found in Creyos cognitive assessment scores between the two groups for all tasks performed (Table 2). However, participants in the intervention group obtained higher scores for six out of nine tasks (‘Feature Match', ‘Double Trouble', ‘Monkey Ladder', ‘Polygons', ‘Spatial Span', and ‘Token Search').

**Table 2:** Creyos cognitive assessment scores of the intervention and placebo group for each task.

**Creyos cognitive assessment tasks (gameplay adjusted)**	**Intervention group**	**Placebo group**	***p-*value**
Feature match	35.9 ± 16.0	34.3 ± 12.1	0.71
Digit span	1.5 ± 0.6	1.6 ± 0.3	0.60
Double trouble	14.6 ± 5.9	12.7 ± 5.2	0.25
Monkey ladder	1.7 ± 0.4	1.6 ± 0.2	0.25
Polygons	14.5 ± 10.2	11.1 ± 6.1	0.19
Rotations	32.9 ± 23.6	33.7 ± 17.8	0.89
Spatial planning	10.9 ± 5.9	11.1 ± 4.0	0.86
Spatial span	1.5 ± 0.6	1.5 ± 0.3	0.84
Token search	1.9 ± 0.4	1.8 ± 0.3	0.36

*p*-values from a test of difference in proportion with an independent t-test. Results for Creyos cognitive assessment tasks were adjusted in difficulty and/or scores to better represent individual performance.

### 3.4 Keyboard and mouse inputs

#### 3.4.1 Keyboard inputs

**Figure 3:**
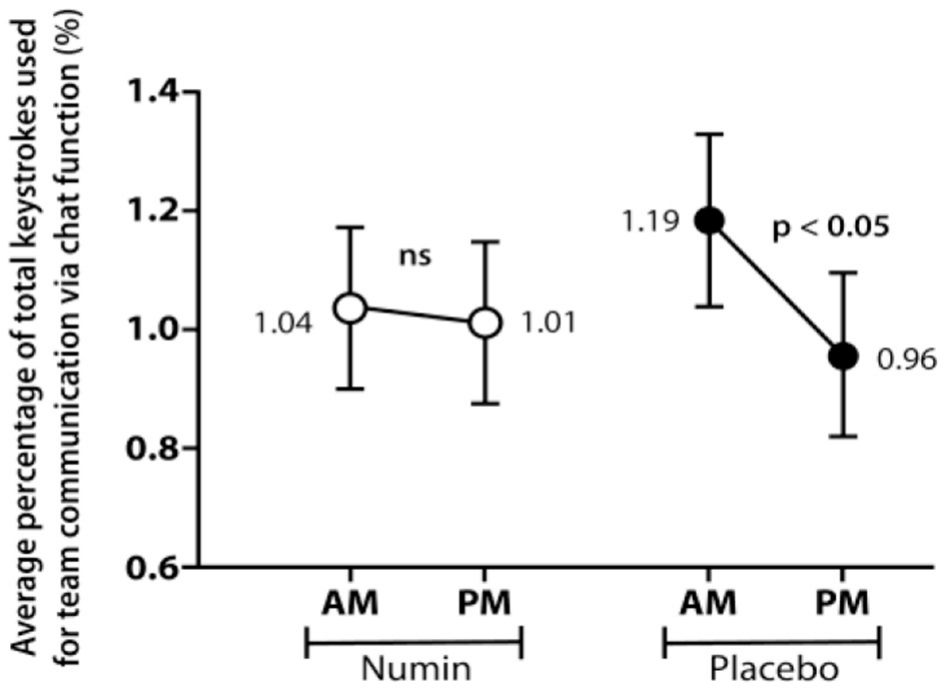
Average chat cycles of participants from morning (AM) to evening (PM) gaming session in the intervention and placebo groups. *p*-value from a test of difference in proportion with a paired *t-*test. *ns* denotes a difference that is not statistically significant.

**Figure 4:**
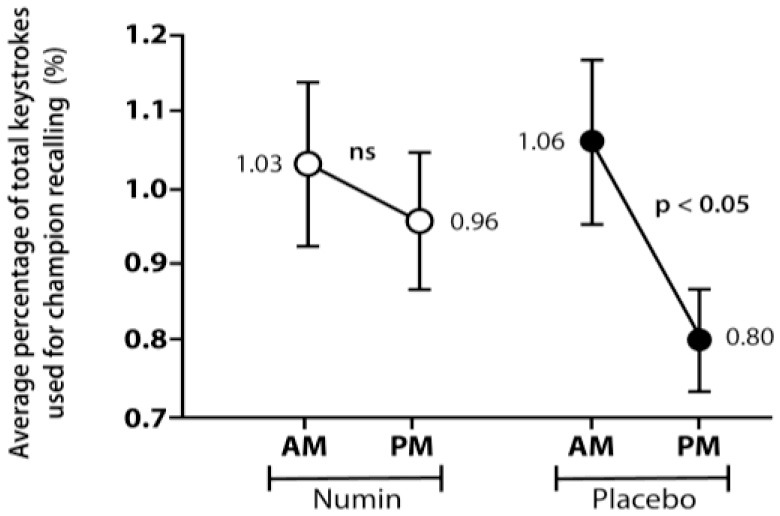
Average recalling of champions by participants from morning (AM) to evening (PM) gaming sessions in the intervention and placebo groups. *p*-value from a test of difference in proportion with a paired *t-*test. *ns* denotes a difference that is not statistically significant.

Figures 3 and 4 depict the chat cycles and champion recalling for both groups across morning (AM) and evening (PM) sessions, respectively. The intervention group (Numin) had no significant difference in chat cycles or champion recalling. However, the placebo group demonstrated significant decreases in both chat cycles (Placebo_AM_ = 1.19, Placebo_PM_ = 0.96, *p* < 0.05) and champion recalling (Placebo_AM_ = 1.06, Placebo_PM_ = 0.80, *p* < 0.05).

#### 3.4.2 Mouse inputs

**Figure 5:**
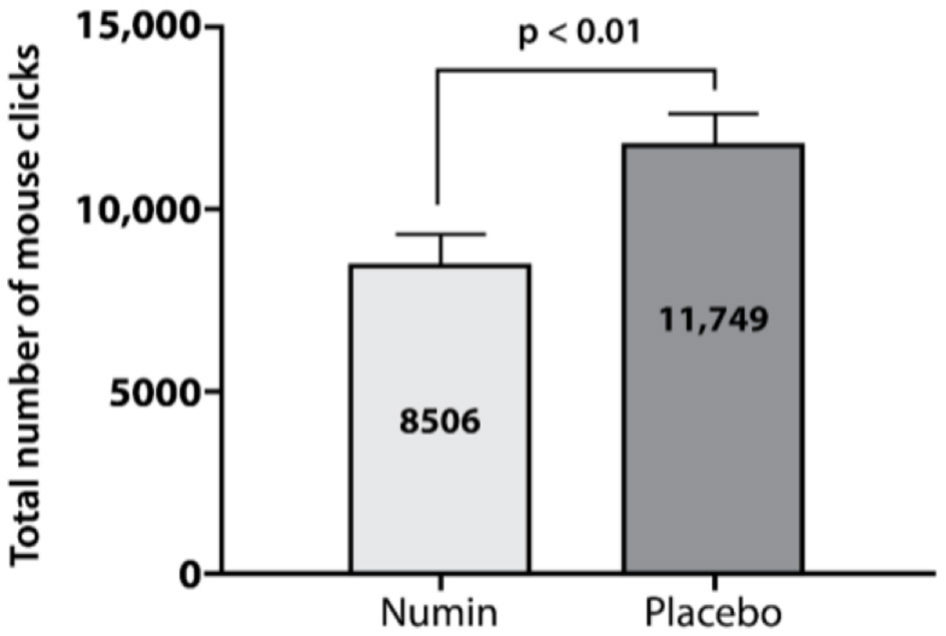
Total number of mouse clicks made by participants across all games in the intervention and placebo groups. *p*-value from a test of difference in proportion with an independent *t*-test.

**Figure 6:**
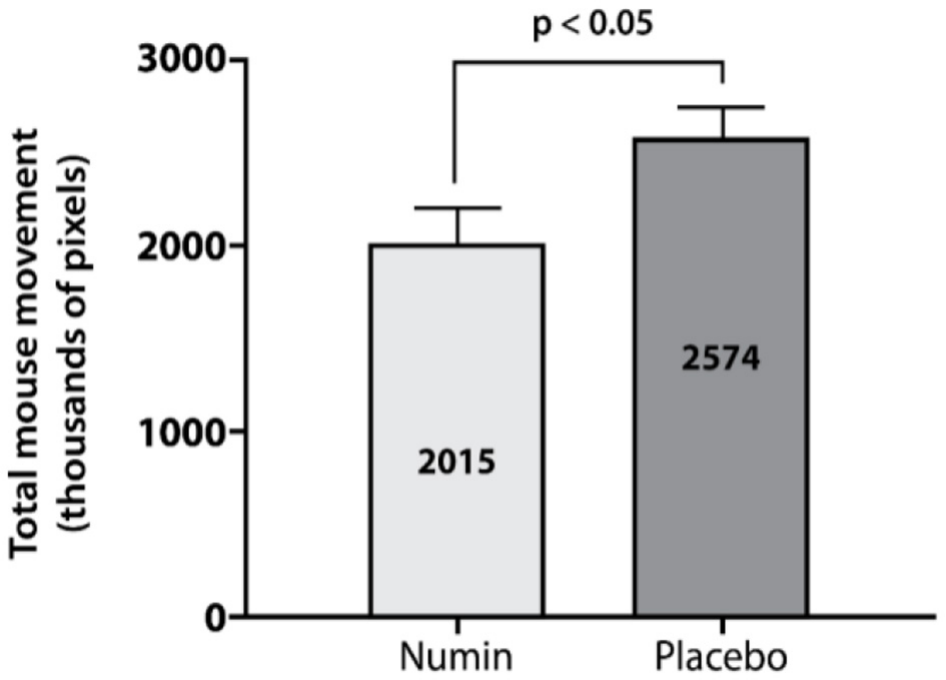
Total mouse movement made by participants across all games in the intervention and placebo groups. *p*-value from a test of difference in proportion with an independent *t*-test.

The intervention group had a significantly lower total number of mouse clicks (Numin = 8,506, Placebo = 11,749, *p* < 0.01) (Figure 5) and total mouse movement in thousands of pixels (Numin = 2,015, Placebo = 2,574, *p* < 0.05) (Figure 6) compared to the placebo group.

### 3.5 Video game performance

#### 3.5.1 Game outcomes

**Figure 7:**
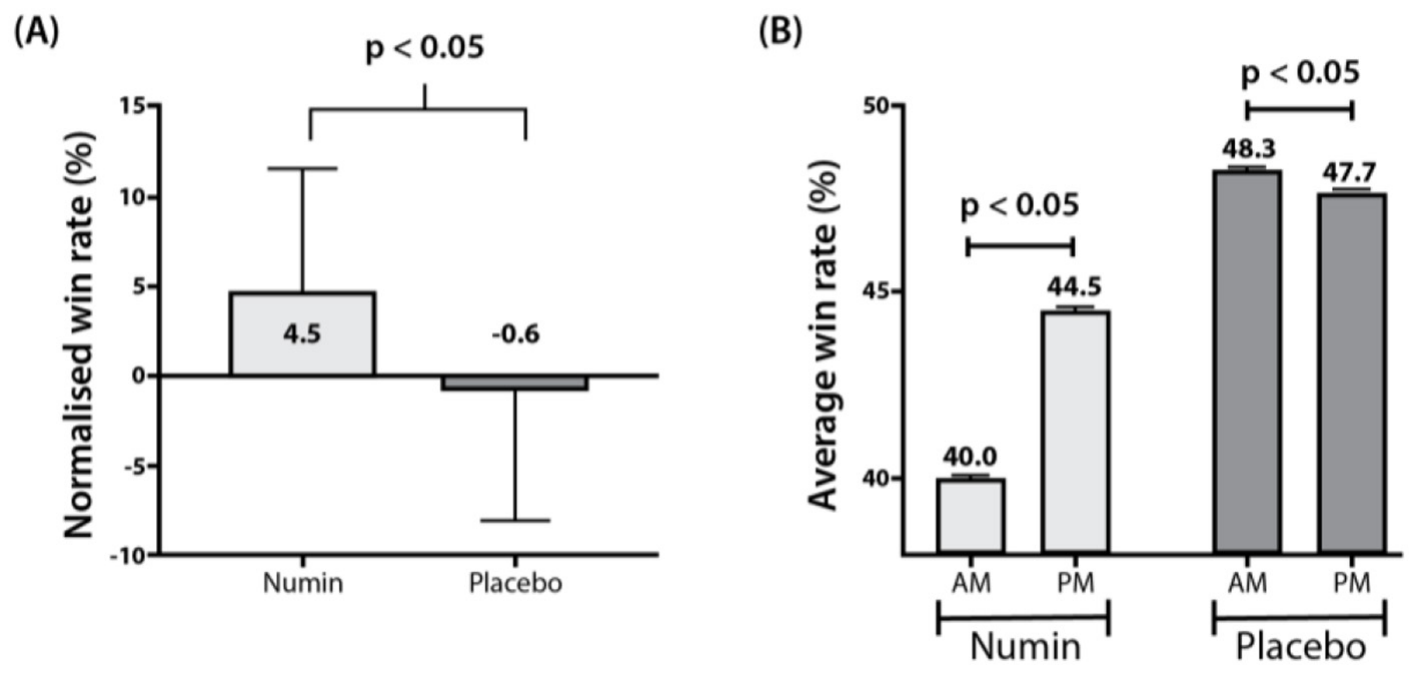
Comparison of win rates between the intervention and placebo groups. **(A)** Normalized win rates of the intervention and placebo group. Normalization was done using each groups' baseline win rate from the morning gaming session. **(B)** Average win rates from morning (AM) and evening (PM) gaming sessions for the intervention and placebo groups. *p*-values from a test of difference in proportion with repeated measures ANOVA.

**Figure 8:**
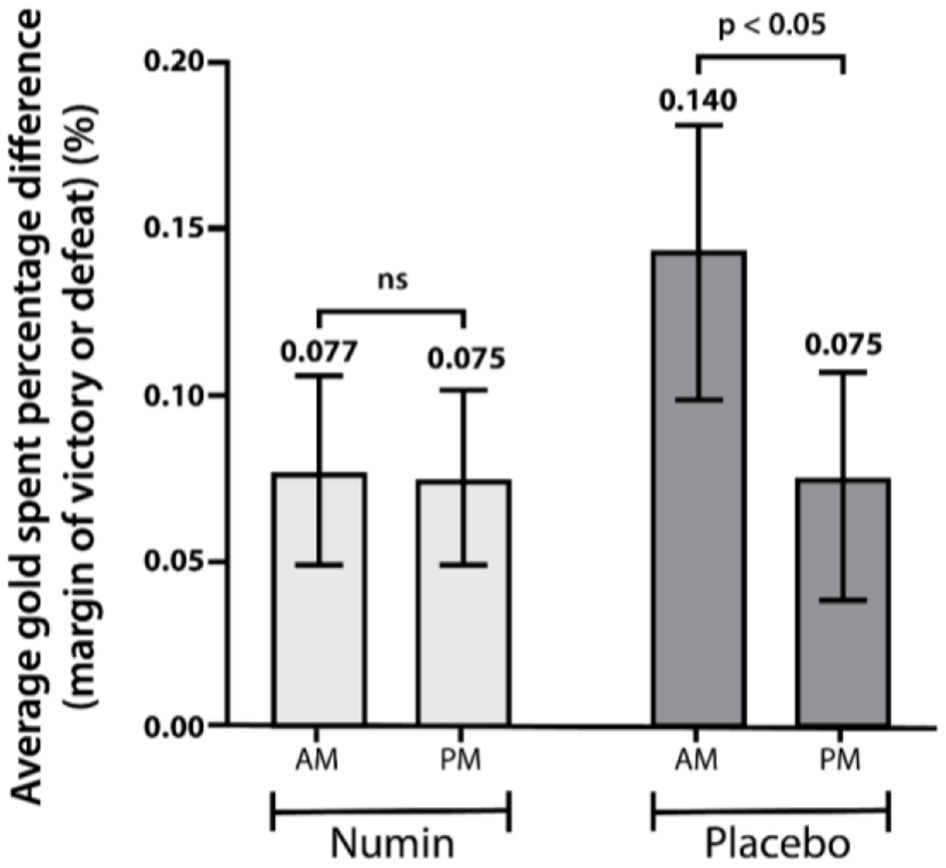
Average gold spent percentage difference (GSPD) of the intervention and placebo groups during morning (AM) and evening (PM) gaming sessions. *p*-value from a test of difference in proportion with repeated measures ANOVA (RM-ANOVA). *ns* denotes a difference that is not statistically significant.

A significant difference was observed in the normalized win rates between the intervention and placebo groups (*p* < 0.05), in which the intervention group saw a 4.5% increase while the placebo group saw a 0.6% decrease (Figure 7A). Furthermore, a significant difference was demonstrated in the win rates within both groups (Numin_AM_ = 40.0, Numin_PM_ = 44.5, *p* < 0.05; Placebo_AM_ = 48.3, Placebo_PM_ = 47.7, *p* < 0.05) (Figure 7B). Regarding the win-loss margin, a significant reduction in GSPD was noted within the placebo group (Placebo_AM_ = 0.140, Placebo_PM_ = 0.075, *p* < 0.05), but not in the intervention group (Figure 8).

#### 3.5.2 Overall player performance

**Figure 9:**
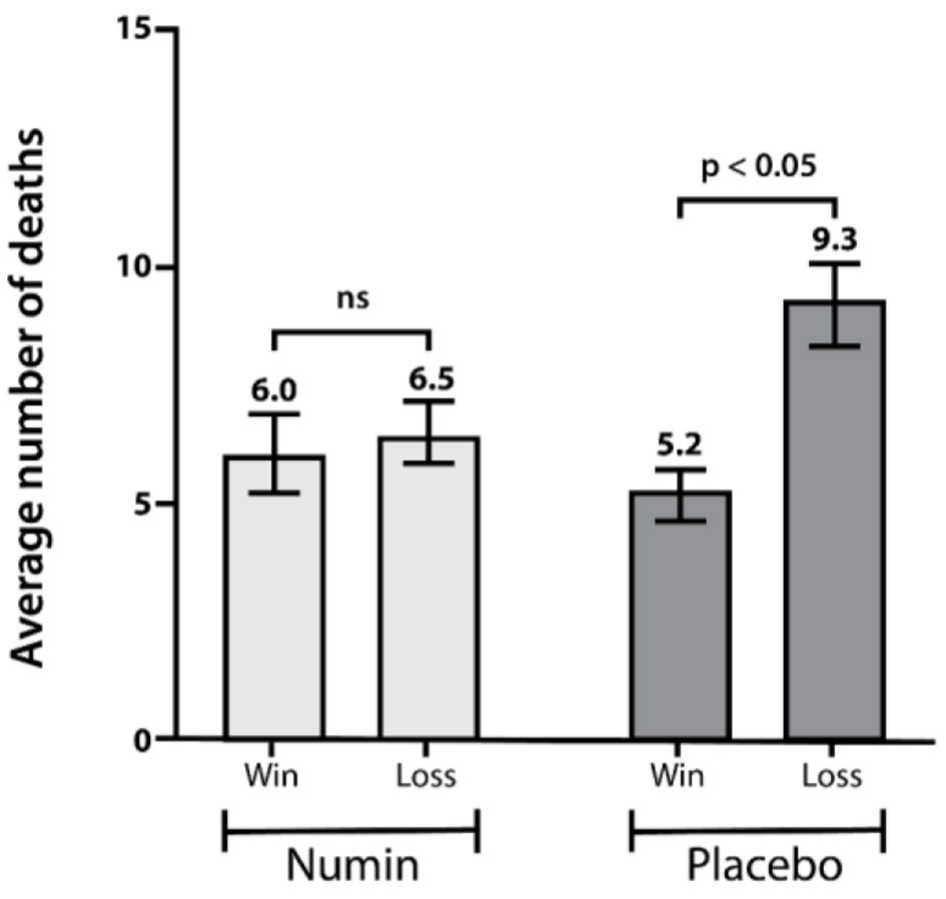
Average number of deaths between the intervention and placebo groups according to game outcomes. *p*-value from a test of difference in proportion with repeated measures ANOVA (RM-ANOVA). *ns* denotes a difference that is not statistically significant.

Figure 9 shows the average number of deaths in both groups according to game outcomes. The number of deaths in the placebo group was significantly higher when the game was lost compared to when it was won (Placebo_Win_ = 5.2, Placebo_Loss_ = 9.3, *p* < 0.05). No significant difference was found in the intervention group.

## 4 Discussion

To our knowledge this is the first study to investigate the efficacy of a multi-nutrient supplement (Numin) in addressing decision fatigue among LoL gamers. The primary endpoint of this study was the change in decision fatigue between the two groups, measured using DFS and video game performance. The secondary endpoints were the changes in cognitive function and physical aspects between the study groups. While the intervention group showed lower Decision Fatigue Scale (DFS) scores and generally higher Creyos cognitive assessment scores compared to the placebo group, these differences were not statistically significant (*p*-value 0.70 for DFS and non-significant for Creyos tasks). Therefore, it is important to note that direct evidence for a statistically significant reduction in perceived decision fatigue or improvement in broad cognitive ability was not established in this pilot study.

Despite the lack of significant differences in subjective fatigue and cognitive assessments, the intervention group demonstrated significant improvements in several objective video game performance metrics. Specifically, the Numin group showed improved average deaths when losing, gold spent percentage difference, and, notably, win rates compared to the placebo group (*p* < 0.05 for all). Furthermore, significant changes were observed in physical aspects of gaming, including the number of mouse clicks and mouse movement, in the intervention group. These objective performance enhancements suggest that the multi-nutrient supplement may have supported the gamers' ability to maintain a higher level of gameplay performance during cognitively demanding tasks. Relating back to our study, a decline in teamwork and collaboration may be observed through the significant difference in GSPD from baseline in the placebo group, but not in the intervention group. Notably, the intervention group also had a 5.1% higher win rate relative to the placebo group, which is indicative of better performance as more games are won (19). Such an increase in win rate can result in the promotion of players into higher ranked skill levels, which are indicative of a player's overall skill and proficiency at the game (20).

Recent work by Leelartapin et al. provides valuable context on the physiological underpinnings of cognitive fatigue in gaming populations. Their study, focusing on military pilot trainees who are gamers, revealed that cognitive fatigue can reduce attention and brain blood flow. Intriguingly, it challenged the common perception that habitual gamers are more tolerant to mental strain, suggesting they might actually be *less* tolerant to cognitive fatigue than non-gamers, potentially due to lower brain oxygen levels. These findings underscore the physiological basis of cognitive fatigue, even in populations accustomed to intense mental demands like gaming (21). The observed improvements in our intervention group, particularly the stable chat cycles and champion recalls, alongside enhanced win rates, suggest that Numin may offer a complementary strategy to improve performance and endurance during cognitively demanding gaming sessions. While Leelartapin et al. pointed to lower brain oxygen levels as a potential factor, our supplement, through its active ingredients like curcumin and *Rhodiola rosea* root extract (RrE) which are linked to neuroprotective properties against glutamate excitotoxicity (22–24), and methylsulfonylmethane's antioxidant effects, could potentially contribute to improved brain metabolic efficiency or protection against neuronal disruption, thereby indirectly supporting better oxygen utilization and increased resilience to cognitive demands.

Numin, administered in the intervention group, appears to improve physical aspects of gaming measured using mouse input mechanics. Our study demonstrates that the placebo group had significantly higher total mouse clicks and total mouse movement compared to the intervention group. These findings point to more erratic mouse movements in the placebo group, whereas the intervention group was more deliberate. This is supported by a study which found that less mouse clicks are associated with more deliberate responding and greater attentiveness while doing computer tasks (25). However, acknowledging that mouse inputs may be influenced by other factors not controlled in this study, such as gameplay strategies and champions selected, future studies should control these variables (26, 27). Nevertheless, these results imply that the intervention possibly induces more deliberate actions, which could contribute to improved performance and consistency during cognitively demanding tasks.

The intervention appears to support performance without a stimulatory effect on cognition, unlike traditional simulants like caffeine which stimulates cognition through competitive antagonism of adenosine in the brain (28). Caffeine is the most common stimulant used by video gamers, claiming its ability to enhance video game performance, concentration, and focus (29, 30). Although caffeine has been shown to stimulate cognitive function (31–33), negative cognitive effects like increased impulsivity, anxiety, and jitteriness can decrease gaming performance (14). In our study, while the intervention group exhibited higher Creyos cognitive assessment scores in most tasks compared to the placebo group, these differences were not statistically significant. This suggests that the intervention's impact on directly measured cognitive functions, as assessed by the Creyos tasks, was not robust in this pilot study. However, the active ingredients present in Numin are still hypothesized to influence neurological pathways relevant to cognitive processing. Glutamate accumulation in the brain is associated with decision fatigue and is known to be excitotoxic to neurons, resulting in the disruption of synaptic transmissions (5). The study intervention contains curcumin and *Rhodiola rosea* root extract (RrE), which have been shown to exert neuroprotective effects through improved glutamate re-uptake, and in certain circumstances, protect against excitotoxic injury (25–27). Therefore, we propose that the intervention used in this study may still support cognitive function and performance during intense tasks through the neuroprotective properties of curcumin and RrE against glutamate excitotoxicity. However, as limited literature exists on the neuroprotective effect of these compounds against glutamate excitotoxicity in this specific context, further research is required to determine their potential role in supporting performance.

Other active ingredients present in the study intervention may confer additional cognitive benefits. For instance, video gamers are associated with an increased risk of oxidative stress, which is known to impede neuronal signaling and may influence decision making (34–36). The antioxidant properties of methylsulfonylmethane are well-documented and may pass through the blood-brain-barrier, potentially mitigating oxidative stress in the brain by suppressing the cellular production of reactive oxygen species (37, 38). This suggests that methylsulfonylmethane may have neuroprotective effects against oxidative stress induced by cognitively demanding tasks. Additionally, studies show that the dietary supplementation of L-tyrosine and chromium picolinate improves dopamine synthesis and enhances serotonin receptor sensitivity, which are important components of signaling systems involved in decision making (3, 39, 40). Therefore, this could imply that L-tyrosine and chromium picolinate enhance the efficiency of neurotransmissions, including those involved in decision making. Although highly speculative and not supported by, for example, any biomarkers, these active ingredients may explain the non-significant 5.13% reduction in decision fatigue seen in the intervention group of our study. All the five active ingredients present in the supplement are likely to cross the blood-brain barrier, but the study did not attempt to isolate the individual effects of each ingredient. Important aspects such as the bioavailability and synergy between the active ingredients were also not investigated in detail. This makes it challenging to attribute the observed effects to specific components or their interactions. The study did not adequately address the role of nutrient absorption and food interactions in modulating the efficacy of Numin. Given the known bioavailability challenges of certain ingredients—particularly curcumin, which is fat-soluble and whose absorption can be altered depending on the fat content of a participant's meals—this aspect warrants greater attention to ensure accurate interpretation of the supplement's effects, even with the use of a high-bioavailability form like TurmiPure Gold^®^ (41). The absence of physiological markers, such as cortisol levels, also limited our ability to objectively assess the biological underpinnings of decision fatigue and the intervention's impact. Future research could benefit from integrating such physiological measurements and more comprehensive dietary assessments to provide a more robust and objective evaluation of the mechanisms of action. There is also a lack of studies that evaluate the effects of Numin's active ingredients on decision making, therefore more research is needed.

A strength of this study is that it is the first, to our knowledge, to assess the effects of a multi-nutrient supplement on decision fatigue in LoL gamers. Additionally, the study cohort was representative of the average skill group of the game, having at least “Silver” rank (42). Participants were also considered well-experienced with a minimum account level of 30, which requires at least 98–119 h of in-game experience (43, 44). However, the generalizability of our findings is limited due to the specific characteristics of our study sample. Participants were a small group of healthy gamers with a “Silver” rank or higher, and notably, the sample included only one female participant. This represents a very specific demographic within the vast gaming population, and the severe gender imbalance may affect the generalizability of the results. Therefore, the findings may not be directly transferable to other gamer populations or individuals experiencing decision fatigue in different contexts. While the study was conducted in an e-sports center, providing a realistic and contextually relevant environment for observations, this specific setting may also limit the generalizability of the findings to less controlled environments, such as participants' homes. Future research could explore the effects of Numin in more varied settings to assess its broader applicability. The relatively small sample size (*N* = 23) impacted the statistical power of the study. While acceptable for a pilot study, it is important to note that this study utilized a crossover design which inherently increases statistical power by reducing inter-individual variability and allows for more precise comparisons. Nevertheless, this limitation may still have contributed to the non-significant differences observed in some measures, such as the Decision Fatigue Scale (DFS) and Creyos cognitive assessment scores, despite the substantial improvements seen in video game performance and physical aspects. Future studies with larger sample sizes are needed to confirm these findings and enhance statistical robustness. Additionally, the video game performance metrics measured in this study did not consider extraneous factors like motivation, sleep, mood, and stress, which are known to affect video game performance, hence considered a limitation of this study (45). While our study focused on healthy adults and, as per our exclusion criteria, did not include participants currently taking prescription medications known to affect cognitive function or energy levels, such as low-dose naltrexone (LDN), we recognize the growing body of research exploring the potential impact of LDN on fatigue, particularly in various chronic conditions. LDN is thought to exert its effects through several potential mechanisms, including modulating the immune system, reducing inflammation, and potentially influencing neurotransmitter systems that play a role in energy regulation and fatigue perception. Studies in conditions like fibromyalgia, chronic fatigue syndrome, and multiple sclerosis have suggested a potential inverse correlation between LDN use and reported fatigue levels (46–48). Given these emerging findings, future research specifically targeting chronic fatigue populations or those with conditions where fatigue is a prominent symptom could significantly benefit from considering the potential relevance of medications like LDN. Such studies might incorporate screening for or controlling for LDN use among participants to better understand its interaction with baseline fatigue levels and the effects of potential interventions. Exploring the interplay between LDN's proposed mechanisms and the physiological pathways underlying fatigue in different populations could provide valuable insights for developing more targeted and effective strategies to manage fatigue.

Specifically, regarding decision fatigue, which was the focus of our study, it is a form of cognitive fatigue that arises from making numerous decisions. While our current study in healthy gamers did not involve LDN, acknowledging its potential role in other contexts is important for a comprehensive understanding of the broader physiological landscape influencing fatigue and informing the design of future research. Future studies investigating decision fatigue in specific chronic populations where LDN is used could explore whether there is an interaction between LDN, the underlying condition, and the experience of decision fatigue.

Regarding the DFS, typically it has been used to measure an individual's decision fatigue over 24 h but was only employed over a 13-h period in this study, potentially limiting its ability to fully capture the effects of decision fatigue (18). The DFS was selected for this study to provide a quantitative, subjective measure of participants' perceived decision fatigue, encompassing emotional, cognitive, and behavioral aspects, including emotional distress, mental exhaustion, and impulsive decision-making. However, it is important to acknowledge that the observed significant improvements in game performance in the intervention group, despite non-significant changes in DFS scores, suggest that the DFS may have been insufficient in capturing the specific form of acute fatigue experienced by gamers over the 13-h study period. Furthermore, the duration and recovery from decision fatigue were not sufficiently addressed. This study primarily captured acute decision fatigue that developed within a single day. However, it did not explore the recovery timeline in either the placebo or intervention groups. A longer assessment period, potentially including a 24-h follow-up, would be beneficial to understand the dynamics of recovery from decision fatigue. Individual variability in experience and recovery from decision fatigue is another aspect that warrants further discussion. While our study observed general trends, it did not adequately explore how factors such as baseline fatigue levels might influence a participant's recovery timeline or their responsiveness to the intervention. It is plausible that participants with higher baseline fatigue could exhibit different recovery patterns or require distinct interventional approaches compared to those with lower baseline fatigue. Future research should endeavor to characterize this individual variability more thoroughly, perhaps by incorporating personalized assessments of fatigue and recovery kinetics, to better understand differential responses to interventions like Numin

Accurately evaluating the precise dosages of Numin's active ingredients in relation to DFS scores in subsequent investigations is critically important. Such granularity would facilitate the elucidation of individual ingredient contributions, such as those from TurmiPure Gold^®^ and Rhodiolife^®^, to the observed modulations in decision fatigue, thereby advancing our understanding of their respective mechanisms of action and optimal concentrations. This methodological refinement is indispensable for establishing robust dose-response relationships, which are foundational for optimizing Numin's formulation to enhance its efficacy. Furthermore, detailed quantitative data on ingredient dosages significantly augments the scientific rigor and reproducibility of research, enabling more precise experimental replication. Beyond the analysis of individual ingredients, a more comprehensive exploration of potential synergistic or antagonistic interactions among varying dosages of Numin's constituents is warranted to fully characterize their collective impact on decision fatigue. Ultimately, this enhanced understanding of ingredients interactions and dose-response characteristics could inform the development of more targeted and efficacious interventions for mitigating decision fatigue.

In the context of this study investigating decision fatigue in LoL gamers, the Mental Fatigue Scale (MFS) and the Fatigue Severity Scale (FSS) could also be considered for future research compared to the Decision Fatigue Scale (DFS), albiet they are not as intentially tailored to measure decision fatigue specifically. The observed significant improvements in game performance in the intervention group, despite non-significant changes in DFS scores, suggest that the DFS was insufficient in capturing the specific form of fatigue experienced by gamers over the 13-h study period. The MFS, with its focus on mental fatigue, aligns closely with the cognitive demands inherent in competitive gaming and would likely be more sensitive to detecting changes in complex in-game behaviors. The FSS, a widely recognized and validated general measure of fatigue severity, offers a broader assessment of fatigue symptoms, including those of a physical nature that may arise from prolonged gaming sessions. Crucially, both the MFS and FSS are designed to capture the subjective experience of fatigue, which directly influences in-game performance, in contrast to the DFS, which primarily assesses the outcome of decision fatigue. Therefore, the integration of these scales in subsequent studies, would facilitate a more sensitive assessment of fatigue within this specific population.

The safety profile of Numin supplement under the study conditions was favorable, with no adverse events (AEs) or serious adverse events (SAEs) reported in either the intervention or placebo groups throughout the study duration (as detailed in the Results and Safety Assessment sections). This underscores the supplement's good tolerability and reinforces its potential as a safe dietary intervention for the target population.

## 5 Conclusions

In conclusion, this pilot study provides preliminary insight into the use of a multi-nutrient supplement to support individuals undergoing cognitively intense tasks, such as video gaming. While the intervention group exhibited a 5.13% lower mean Decision Fatigue Scale (DFS) score compared to the placebo group, this difference was not statistically significant. However, the intervention group did show substantial improvements in video game performance and physical aspects of gaming, demonstrating more consistent decision-making compared to the placebo group. The Numin supplement's active ingredients, including TurmiPure Gold^®^ and Rhodiolife^®^, are believed to contribute to neuroprotective effects. These may involve enhancing glutamate re-uptake and offering protection against excitotoxic injury, thereby fostering optimal neuronal health and neurotransmission. Other active ingredients, such as methylsulfonylmethane, L-tyrosine, and chromium picolinate may also preserve and enhance neurotransmissions involved in decision making. These findings highlight the potential applications of the intervention not only in the e-sports industry, but also in addressing the burden of decision fatigue among individuals who routinely make consecutive critical decisions (49–52).

## Data Availability

The original contributions presented in the study are included in the article/supplementary material, further inquiries can be directed to the corresponding author.

## A Appendix A

**Table A1:** Constituents of the intervention and placebo used in this study.

	**Intervention (Numin)**	**Placebo**
**Active ingredients**	Methylsulfonylmethane, L-Tyrosine, Rhodiolife^®^ (Rhodiola rosea (roots), total rosavins ≥ 5%, salidroside ≥ 1.8%), TurmiPure Gold^®^ (curcuminoids > 30%), Chromium Picolinate	N/A*
**Inactive ingredients**	Xantham Gum, Beet Powder, Stevia, Citric Acid, Natural Orange Passionfruit Flavor	Xantham Gum, Turmeric Color Powder, Beet Powder, Calcium Carbonate, Stevia, Citric Acid, Maltodextrin, Natural Orange Passionfruit Flavor

*Not applicable (N/A).
